# Information Practice as Dialogue: The Case for Collaboration in Evidence Searching and Finding for More Complex Reviews

**DOI:** 10.1002/cesm.70029

**Published:** 2025-04-28

**Authors:** Parkhill Anne, Merner Bronwen, Ryan Rebecca

**Affiliations:** ^1^ Cochrane Consumers and Communication, Centre for Health Communication and Participation, Department of Public Health La Trobe University Melbourne Australia; ^2^ Melbourne School of Population and Global Health University of Melbourne Melbourne Australia

## 1 Background

Cochrane Consumers and Communication Group's (CCC) approach to evidence searching has evolved over time in the context of Cochrane's rigorous methodological advice [1, 2]. CCC is a Cochrane review group responsible for coordinating the preparation and publication of evidence syntheses that affect the way people interact with healthcare professionals, services and researchers. CCC includes a highly skilled Information Specialist who collaborates with CCC author teams to design a rigorous search strategy to gather evidence to answer the review question. In this commentary, we discuss the transformation of the information practice of searching in CCC from being a largely technical exercise conducted solely by the Information Specialist to a collaborative dialogue between the Information Specialist and author teams.

A key reason for the transformation in our search methods has been that CCC reviews tend to be complex, with review questions that are generally not as easily answered as clinically focused reviews. Our research, and information practice specifically, is contextualized and guided by a three‐way dynamic of patient preferences and experiences, research evidence, and professional expertize. The reviews are rigorous in their examination of evidence on people's healthcare interactions, including how people self‐manage health and disease, understand screening, health and treatment, and negotiate and share decisions with healthcare professionals within systems and different settings. However, interventions to change behaviors, to educate, support and up‐skill people to participate actively in their healthcare, are often complex, multifaceted and their effects evaluated via multiple diverse outcomes [3]. This complexity necessarily shapes our methods of information practice.

Early in the life of CCC and for many years, we viewed searching as a largely solitary technical exercise performed by a skilled Information Specialist following conventional, rigorous Cochrane search methods. Often this required labor‐intensive search development, resulting in delays for search results and an excessive screening obligation (e.g., some review questions resulted in authors needing to screen more than 25,000 search results). As volume and complexity of literature in the health communication area increased, we moved towards search strategies developed with practicalities of reference screening in mind [4, 5]. We have since developed transparent and pragmatic search strategies by means of embedded and open dialogue [6] with authors. In the context of increasing topic complexity and rigorous information searching, this approach maximizes identification of relevant references while avoiding unmanageable reference numbers for screening.

In this commentary, we explore CCC's approach to searching and its evolution over time in the context of Cochrane's rigorous methodological advice. We illustrate different approaches to achieving this balance between rigour and the practical demands of review production. To demonstrate our approach, we will discuss two recent CCC reviews [7, 8] that showcase the development of our current practices.

### 1.1 Pragmatic, Joint Decisions for Effective Searching

The growth in eligible research for complex communication reviews has been accompanied by real‐time developments in our Group's author collaboration. We encourage and facilitate discussion with authors of the most appropriate search process and in developing the answerable question(s), as advised by Cochrane [9]. This collaboration between authors and the Information Specialist has the additional benefit of helping authors clarify how they will structure and address the issues in the planned review. A flexible, two‐way process results and this underpins the cost–benefit of time spent on the proposed search method instead of following a standard search formula for all Cochrane review types [4].

Our decision concurs with Cochrane's advice that “review authors should work closely, from the start of the protocol, with an experienced medical/healthcare librarian or Information Specialist” [2]. This advice for dialogue underpins and makes our information‐gathering processes successful [1]. With the inclusion of new sources for Qualitative Evidence Syntheses (QES), we have moved beyond the PICO search structure to adopt other search structures or mnemonics that form the framework for strategy development and study screening [1].

#### 1.1.1 Example Review

What are relevant, feasible and effective approaches to promote acceptance, uptake and adherence to physical distancing measures for COVID‐19 prevention and control [8]?

In the rush of COVID‐19 in early 2020, CCC was commissioned by the World Health Organization (WHO) to review rapidly the evidence on approaches to promote acceptance, uptake and adherence to physical distancing measures for COVID‐19 [8]. Consultations with WHO informed initial scoping by the author team. This scoping showed relevant literature as extremely broad in content and methods, as COVID‐19‐specific research was only emerging. Therefore, working with the author team, we developed a search strategy comprising two Core term sets (“Communicable diseases“ and “Health communication”). The combination of the two Core sets was further filtered by the six non‐pharmaceutical interventions (NPIs) of interest to WHO (e.g., “quarantine,” “crowd avoidance,” and “isolation”) [8].

Our search method tended toward specificity rather than sensitivity; each term in the Core and NPI sets was checked rigorously to ensure that the retrieved references were specific and relevant. Based on methods reported in previous research, we closely evaluated the choice of terms for strategies [4].

We stopped searching once relevant references generated from weekly searches had decreased in number, a decision reached jointly by the Information Specialist and senior members of the research team, although a more conventional Cochrane strategy would have drawn a wider net. The search refinement ensured that identified references were at a number that proved manageable for continuous screening for 6 weeks, despite not being limited by study design, and in a total review time of 10 weeks.

This search approach was the basis of an update recently published as a Cochrane Rapid Review [10]. The significant proliferation of COVID‐19 papers in the short period between the two review versions necessitated further refinement of search methods based on dialogue with the author team.

### 1.2 Evidence Sources Informed by Each Unique Search Question

Search results can be streamlined by choosing from the recommended source databases and websites with a strategic eye for maximal recall. As part of the search process, the initial draft search strategy is tested across various electronic databases, including those required by Cochrane standards [2]. This method also aims to find those additional databases most likely to retrieve relevant numbers of unique references. Searching two or more databases reduces the risk of missing relevant studies [11], particularly for qualitative research [12]. However, the number of references to screen can easily grow to unmanageable numbers if too many databases are chosen. We consider no value is gained by searching electronic databases merely because we have used them in the past. Instead, we work to identify systematically the optimal sources of unique references for each review.

A previous report of sources of included studies used in guideline development [13] showed that electronic databases were but one of many productive sources. The work of searching is, therefore, not only to identify the optimal electronic database sources but also to identify other sources of relevant evidence (e.g. reports, dissertations, organizational websites and conference abstracts, Google Scholar), without which search results for comprehensive reviews may be incomplete [1]. Regardless of the search question, we check whether relevant included references that the authors have already identified or those that are found in preliminary scoping searches are retrieved in the draft searches. This reality‐checking against references likely to meet the review's inclusion criteria (validation references) builds confidence in the final strategies.

#### 1.2.1 Example Review

Consumers' and health providers' views and perceptions of partnering to improve health services design, delivery and evaluation: a co‐produced qualitative evidence synthesis [7].

Database choice was particularly relevant for this recently co‐produced QES as the topic, perceptions of consumer‐health provider partnering in health services, was extremely broad, cross‐disciplinary, and lacking consensus on key definitions. The lead author and Information Specialist worked closely to develop a search strategy that retrieved both the 10 identified validation references and a manageable number of total references for screening. The following paragraph illustrates that decisions arising from our dialogue balance pragmatism with rigorous methodology.

During reference list checking of the included studies identified via database searches, we found that some relevant articles had not been retrieved. A further set of five validation references, compiled from the articles found from reference list checking, was run through the databases used for the database searching (MEDLINE, Embase, CINAHL, PsycINFO). As none of the individual databases included all five articles, this required more exploration. For example, we determined that the further five validation references were indexed in Scopus but that a Scopus search would be too broad‐ranging and retrieve many irrelevant references. Similarly, we abandoned a plan to refine the CINAHL search strategy because we determined that this database did not index all five references we identified as relevant evidence for the review. By working collaboratively and iteratively through databases, we, therefore, decided that the combination of the existing database searches and reference list checking would be sufficiently rigorous when balanced against reference numbers. We ultimately chose to search four databases, as well as searching the reference lists of included studies, for added assurance.

## 2 Conclusions

Developments of the evidence‐based practice (EBP) process and methods in the last 30 years have added nuance and depth to collective knowledge in healthcare. For the EBP methods to remain gold standard and useful, researchers and Information Specialists have added layers of procedural sophistication at all steps of the EBP chain. Information Specialists bring unique skills to the field of evidence synthesis and there is growing recognition of the value of their contribution in identifying and managing large and diverse evidence sources [14, 15]. Search, our example here, has moved from being modeled on a basic PICO framework run primarily in databases to one of increasingly sophisticated choices between evidence sources and search terms to describe and inform the concepts contained within search questions.

Search now calls for dialogue with interest holders. This can be managed by the Information Specialist, whose task is to balance the often complex concepts against the time and effort required to address the answerable search question(s). To achieve this dialogue, Information Specialists must be integrated and embedded into the processes of review production [14, 16]. We have found that only through iterative testing and dialogue can we achieve rigorous but manageable search results in an effective balance of the art with the science of evidence retrieval.

## Author Contribution

**Parkhill Anne:** conceptualization, investigation, writing − original draft, writing − review and editing, project administration, funding acquisition. **Merner Bronwen:** conceptualization, writing − original draft, writing − review and editing, project administration, investigation, funding acquisition. **Ryan Rebecca:** conceptualization, investigation, writing − original draft, writing − review and editing, project administration, supervision, funding acquisition.

## Conflicts of Interest

The authors declare no conflicts of interest.

## 1 Peer Review

The peer review history for this article is available at https://www.webofscience.com/api/gateway/wos/peer-review/10.1002/cesm.70029.

## Data Availability

The authors have nothing to report.

## References

[1] J. L. Harris , A. Booth , M. Cargo , et al., “Cochrane Qualitative and Implementation Methods Group Guidance Series‐Paper 2: Methods for Question Formulation, Searching, and Protocol Development for Qualitative Evidence Synthesis,” Journal of Clinical Epidemiology 97 (2018): 39–48, 10.1016/j.jclinepi.2017.10.023. 29248725

[2] C. G. J. Lefebvre , S. Briscoe , R. Featherstone , et al., “Chapter 4: Searching for and Selecting Studies,” in Cochrane Handbook for Systematic Reviews of Interventions version 64 (updated October 2023), eds. J. P. T. Higgins , J. Chandler , M. Cumpston , T. Li , M. J. Page , and V. A. Welch , (Cochrane, 2023), https://www.training.cochrane.org/handbook.

[3] T. Greenhalgh , D. Fisman , D. J. Cane , M. Oliver , and C. R. Macintyre , “Adapt or Die: How the Pandemic Made the Shift From EBM to EBM+ More Urgent,” BMJ Evidence‐Based Medicine 27, no. 5 (2022): 253–260, 10.1136/bmjebm-2022-111952. PMC9510422 35853682

[4] A. F. Parkhill , O. Clavisi , L. Pattuwage , et al., “Searches for Evidence Mapping: Effective, Shorter, Cheaper,” Journal of the Medical Library Association: JMLA 99, no. 2 (2011): 157–160, 10.3163/1536-5050.99.2.008. 21464854 PMC3066574

[5] Y. Fu , Y. Mao , S. Jiang , S. Luo , X. Chen , and W. Xiao , “A Bibliometric Analysis of Systematic Reviews and Meta‐Analyses in Ophthalmology,” Frontiers in Medicine 10 (2023): 1135592, 10.3389/fmed.2023.1135592. 36936241 PMC10017479

[6] G. Gerard and L. Ellinor . Flexing a Different Conversational “Muscle”: The Practice of Dialogue Dana, California: Leverage Networks; 2018, https://thesystemsthinker.com/flexing-a-different-conversational-muscle-the-practice-of-dialogue/.

[7] B. Merner , L. Schonfeld , A. Virgona , et al., “Consumers' and Health Providers' Views and Perceptions of Partnering to Improve Health Services Design, Delivery and Evaluation: A Co‐Produced Qualitative Evidence Synthesis,” Cochrane Database of Systematic Reviews 3 (2023): 013274, 10.1002/14651858.CD013274.pub2. PMC10065807 36917094

[8] R. E. Ryan , A. Parkhill , L. Schonfeld , et al., What Are Relevant, Feasible and Effective Approaches to Promote Acceptance, Uptake and Adherence to Physical Distancing Measures for COVID‐19 Prevention and Control?: World Health Organization, Regional Office for Europe; 2021, https://www.ncbi.nlm.nih.gov/books/NBK571247/. 34152715

[9] M. Cumpston and E. Fleming , “Chapter II: Planning a Cochrane Review,” in Cochrane Handbook for Systematic Reviews of Interventions version 64 (updated August 2023) [Internet] (Cochrane, 2023), https://www.training.cochrane.org/handbook.

[10] R. E. Ryan , C. Silke , A. Parkhill , et al., “Communication to Promote and Support Physical Distancing for COVID‐19 Prevention and Control,” Cochrane Database of Systematic Reviews 2023, no. 10 (2023): CD015144, 10.1002/14651858.CD015144. PMC10561351 37811673

[11] H. Ewald , I. Klerings , G. Wagner , et al., “Searching Two or More Databases Decreased the Risk of Missing Relevant Studies: A Metaresearch Study,” Journal of Clinical Epidemiology 149 (2022): 154–164, 10.1016/j.jclinepi.2022.05.022. 35654269

[12] T. F. Frandsen , F. A. Gildberg , and E. B. Tingleff , “Searching for Qualitative Health Research Required Several Databases and Alternative Search Strategies: A Study of Coverage in Bibliographic Databases,” Journal of Clinical Epidemiology 114 (2019): 118–124, 10.1016/j.jclinepi.2019.06.013. 31251982

[13] A. Parkhill and K. Hill , “Identifying the Effective Evidence Sources to Use in Developing Clinical Guidelines for Acute Stroke Management: Lived Experiences of the Search Specialist and Project Manager,” Health Information & Libraries Journal 26, no. 1 (2009): 47–55, 10.1111/j.1471-1842.2008.00784.x. 19245643

[14] J. Logan , “Why Do Researchers Co‐Author Evidence Syntheses With Librarians? A Mixed‐Methods Study,” Research Synthesis Methods 14, no. 3 (2023): 489–503, 10.1002/jrsm.1629. 36808812

[15] E. R. Stevens and G. Laynor , “Enhancing the Quality and Efficiency of Regulatory Science Literature Reviews Through Innovation and Collaboration With Library and Information Science Experts,” Frontiers in Medicine 11 (2024): 1434427, 10.3389/fmed.2024.1434427. 39021816 PMC11251899

[16] S. Young , H. MacDonald , D. Louden , et al., “Searching and Reporting in Campbell Collaboration Systematic Reviews: A Systematic Assessment of Current Methods,” Campbell Systematic Reviews 20, no. 3 (2024): e1432, 10.1002/cl2.1432. 39176233 PMC11339316

